# A Co-Culture Model of IPEC-J2 and Swine PBMC to Study the Responsiveness of Intestinal Epithelial Cells: The Regulatory Effect of Arginine Deprivation

**DOI:** 10.3390/ani11092756

**Published:** 2021-09-21

**Authors:** Roberta Saleri, Paolo Borghetti, Francesca Ravanetti, Melania Andrani, Valeria Cavalli, Elena De Angelis, Luca Ferrari, Paolo Martelli

**Affiliations:** Department of Veterinary Science, University of Parma, Strada del Taglio 10, 43126 Parma, Italy; roberta.saleri@unipr.it (R.S.); francesca.ravanetti@unipr.it (F.R.); melania.andrani@unipr.it (M.A.); valeria.cavalli@unipr.it (V.C.); elena.deangelis@unipr.it (E.D.A.); luca.ferrari@unipr.it (L.F.); paolo.martelli@unipr.it (P.M.)

**Keywords:** IPEC-J2, PBMC, co-culture system, arginine, swine

## Abstract

**Simple Summary:**

The interest in amino acids comes from their involvement in research on alternative strategies for the utilization of antibiotics on farms. Among several substances used to replace antibiotics, there is arginine, an essential amino acid in newborns and piglets. This amino acid has a protective role in intestinal immune cells and improves intestinal immunity. The purpose of this research was to define a co-culture model, in which intestinal epithelial cells can communicate with peripheral blood mononuclear cells (PBMC) to deepen the effects of arginine deprivation on intestinal epithelial cells over time. The main finding was that the lack of arginine highly impacts on intestinal and immune cells by way of immuno-regulation mediated by the expression of pro- and anti-inflammatory cytokines. The use of this experimental model could allow us to investigate the impact of and interactions between specific nutrients and the complex intestinal environment and, in addition, to assess feed additives to improve health and animal production.

**Abstract:**

Arginine is a semi-essential amino acid, supplementation with which induces a reduction of intestinal damage and an improvement of intestinal immunity in weaned piglets, but the mechanism is not yet entirely clear. The aim of this study was to characterise a co-culture model by measuring changes in gene expression over time (24 and 48 h) in intestinal IPEC-J2 cells in the presence of immune cells activated with phytohemagglutinin and, consequently, to assess the effectiveness of arginine deprivation or supplementation in modulating the expression of certain cytokines related to the regulation of intestinal cells’ function. The main results show the crucial role of arginine in the viability/proliferation of intestinal cells evaluated by an MTT assay, and in the positive regulation of the expression of pro-inflammatory (*TNF-α*, *IL-1α*, *IL-6*, *IL-8*) and anti-inflammatory (*TGF-β*) cytokines. This experimental model could be important for analysing and clarifying the role of nutritional conditions in intestinal immune cells’ functionality and reactivity in pigs as well as the mechanisms of the intestinal defence system. Among the potential applications of our in vitro model of interaction between IEC and the immune system there is the possibility of studying the effect of feed additives to improve animal health and production.

## 1. Introduction

The gastrointestinal tract (GIT) is constantly exposed to a variety of potentially harmful antigens and molecules [1]. Intestinal epithelial cells (IECs) play a crucial role in the defence of the host’s intestinal mucosa as a physical barrier and, above all, acting as a part of the innate and adaptive immune response. The integrity of the physical barrier is critical for its function: it selects the paracellular and intercellular transit of molecules in the gut and selective nutrient absorption by means of a complex network of tight junctions (TJ) and gap junctions connecting the IECs [2]. In the same way, IECs positively influence the development and homeostasis of the mucosal immune cells with a fundamental immuno-regulatory function [3]. The “gut health” concept [4] includes physiological and functional aspects related to the maintenance of intestinal homeostasis: the focus is on the communication and interaction between these components; therefore not only the digestion and absorption of nutrients but also the immune mechanisms of defence. The close interaction and communication between IECs and immune cells in the lamina propria regulate the gastro-intestinal immune responses [5]. The response to an infection triggers the activation of different responsive cells and the production of signalling molecules, especially pro-inflammatory cytokines such as tumour necrosis factor (TNF)-α, interleukin (IL)-1 and IL-6. IECs also induce resident dendritic cells (DC) to release immune mediators, such as the anti-inflammatory cytokine transforming growth factor (TGF)-β [6]. In swine, the functionality of the gastrointestinal–neuroendocrine axis is strongly connected with feed efficiency, energy balance and growth, in other words with the productive performances [7,8]. An optimally functioning gastrointestinal tract is of importance to the overall metabolism, physiology, disease response and performance of pigs at all stages of growth and development. The interactions between food components, microbiota and immune mediators at the intestinal epithelium are far from being clarified in this species. Furthermore, due to the anatomical and physiological analogies to humans, the pig represents a model of interest for research on human health [9,10]. The maintenance and growth of the intestine is contingent upon the amount and type of nutrients in the lumen [11]; among these different nutrients, arginine has been identified as a modulator of growth performance in weaned piglets [12] due to its direct effects on the intestinal barrier and immune function [13]. L-arginine is an essential amino acid synthesized by enterocytes that plays different physiological roles in protein synthesis, cell proliferation and immune responses [14]. In our study, an in vitro approach using the IPEC-J2 cell line was chosen to investigate some aspects of a complex structure such as the intestinal barrier. IPEC-J2, originated from the epithelium of a pre-colostral pig, is an untransformed permanent intestinal cell line. These cells are morphologically and functionally similar to primary intestinal epithelial cells [15]. In fact, IPEC-J2 show microvilli and tight junctions, and express cytokines, Toll-like receptors and mediators involved in the immune response. All of these characteristics make this line a suitable model for assessing and studying the direct effects of different stimuli. Aiming to develop a model resembling, as much as possible, the interplay between IECs and immune cells, we used a co-culture system with IPEC-J2 and peripheral blood mononuclear cells (PBMC). 

On this basis, the target of this work was firstly to characterize the co-culture model by evaluating the changes in IPEC-J2 gene expression in the presence of activated PBMC and, secondly, to evaluate the effectiveness of arginine in modulating the expression of some cytokines connected to the regulation of intestinal cells’ functions.

## 2. Materials and Methods

### 2.1. Reagents

Dulbecco’s Modified Eagle Medium/Ham’s F-12 (DMEM/Ham’s F-12) medium (standard formulation containing arginine at 147.5 mg/L), Histopaque-1077^®^ solution, penicillin/streptomycin/amphotericin B, glutamine, Trypan blue, 3-(4,5-dimethylthiazol-2-yl)-2,5-diphenyltetrazolium bromide (MTT), dimethyl sulfoxide (DMSO), DNAse, Griess reagent, and phytohemagglutinin (PHA) were purchased from Sigma-Aldrich (St. Louis, MO, USA); foetal bovine serum (FBS) and TRI-reagent were obtained from ThermoFisher (Carlsbad, CA, USA); DMEM/Ham’s F-12 without arginine was obtained from US Biological Life Science (Salem, MA, USA). Oligo-dT primers were purchased from Bioneer (Daejeon, Korea). A high-capacity cDNA Reverse Transcription kit, and Fast Power-Up SYBR Green Master Mix were obtained from Applied Biosystems (Foster City, CA, USA), and primer sets from Eurofins Genomics (Ebersberg, Germany); IPEC-J2 were kindly provided by Dr. A. Baldi (Department of Veterinary Science for Health, Animal Production and Food Safety, University of Milan, Lodi, Italy).

### 2.2. Cell Cultures and Culture Conditions

For the co-culture assessment, two cell types were used: 

(1) porcine peripheral blood mononuclear cells (PBMC);

(2) non-transformed cell line IPEC-J2.

Porcine PBMC were obtained from the blood of adult 9–10 month old pigs from a slaughterhouse certified by the Italian Ministry of Health according to the Regulation (EC) 853/2004 (Sassi S.P.A., Parma, Italy; approval nr. CE-IT-190-M). PBMC were isolated from 4 to 5 mL of blood by density gradient centrifugation with Histopaque-1077^®^ solution. Cells were washed twice with sterile PBS + 1% FBS, immediately frozen at −80 °C using a Mr. Frosty^®^ (Sigma, St. Louis, MO, USA) device and stored in liquid nitrogen until use. IPEC-J2 cells were cultured in DMEM/Ham’s F-12 + 5% FBS, supplemented with 5% penicillin/streptomycin/amphotericin B, glutamine (2 mM) and cultivated in a humidified environment at 37 °C, 5% CO_2_. Cells were used between passages 28–30. The number of IPEC-J2 cells was determined using a haemocytometer and cell viability (never less than 95%) was assessed with Trypan blue (0.1%) exclusion. Before the treatment, viability/proliferation was investigated in a complete and arginine-free medium by using an MTT colorimetric assay. In the assay, the conversion of MTT into a dark violet, water-insoluble formazan was catalysed by mitochondrial dehydrogenases of living/proliferating cells. Briefly, IPEC-J2 cells were seeded in 96-well plates at a density of 10,000 cells/well for 24 and 48 hours (h) with 200 μL of complete culture medium and arginine-free medium. MTT assays were performed by incubating the IPEC-J2 cells with 20 μL (5 mg/mL) of MTT solution. After 4 h of incubation, the medium and MTT solution were removed from the wells, then the IPEC-J2 cells were lysed with 150 μL DMSO, and the purple formazan crystals were solubilized for detection at 490 nm by using a Victor-3^™^ 1420 Multilabel Counter (PerkinElmer, Waltham, MA, USA). Regarding PBMC, after incubation for 24 h with PHA (5 μg/mL), cells were centrifuged and supernatants were discharged. Cells were washed twice with DMEM/Ham’s F-12, resuspended in the same medium or in the arginine-free medium and plated at 2 × 10^5^ cells/well in 96-well plates and incubated for 24, 48 or 72 h. During the last 3 h, 10 μL of 5 mg/mL MTT in sterile PBS was added. After incubation, 100 μL of 0.01 N HCl + 10 % sodium dodecyl sulphate (SDS) was added to develop the reaction. After overnight incubation, plates were read using a Victor-3^™^ 1420 Multilabel Counter (PerkinElmer, Waltham, MA, USA) at 540 nm.

### 2.3. Transwell Co-Culture Conditions

One day before the start of the experiments, PBMC were thawed and viability was determined by Trypan blue exclusion (95%). PBMC were seeded in 24-well tissue culture plates at a density of 6 × 10^6^ cells/well and PHA (5 μg/mL final) was added to 1 mL of DMEM/Ham’s F-12. At the end of PHA incubation, plates were centrifuged, the medium was discharged and 1 mL of DMEM/Ham’s F-12 or DMEM/Ham’s F-12 without arginine was added. Leucine and Lysine were added to the medium according to the formulation of the DMEM/Ham’s F-12. After 24 h, IPEC-J2 were seeded on the top surface of collagenized cell culture inserts (0.3 cm^2^ polyethylene terephthalate membrane with 0.4 μm pore size; Costar, Corning Inc., Corning, NY, USA) at a density of 1 × 10^5^ cells/cell culture insert. The inserts were put into the 24-well plates where PBMC were seeded and 0.5 mL of fresh medium with or without arginine was added to the respective wells. 

The experimental groups, incubated for 24 or 48 h, were as follows:

1. IPEC-J2 monoculture in DMEM/Ham’s F-12 medium, containing arginine (IPEC);
2. IPEC-J2 monoculture in DMEM/Ham’s F-12 without arginine (IPEC/–Arg);
3. IPEC-J2 co-culture with PBMC in DMEM/Ham’s F-12, containing arginine (IPEC+PBMC);
4. IPEC-J2 co-culture with PBMC in DMEM/Ham’s F-12 without arginine (IPEC+PBMC/–Arg).

To clarify the role of PBMC involvement in IPEC-J2 responsiveness, we added the following groups:

5. PBMC monoculture in DMEM/Ham’s F-12, containing arginine (PBMC);
6. PBMC monoculture in DMEM/Ham’s F-12 without arginine (PBMC/–Arg).

At the end of both incubation time-points (24 and 48 h), the viability of IPEC-J2 and of PBMC was evaluated for mono-culture vs. co-culture under experimental conditions (presence/absence of arginine) by Trypan blue exclusion. For each cell type (IPEC-J2 or PBMC), no significant differences were detected with regards to the same cultural and experimental conditions at each time-point.

### 2.4. Nitric Oxide Assay

NO production was assessed by measuring the amount of nitrite (NO_2_^−^), a stable metabolic product of NO. It was measured in the culture medium from cells by Griess reaction after 24 and 48 h of culture. Briefly, 100 μL of cell culture medium was mixed with 100 μL of Griess reagent [equal volumes of 1 % (*w*/*v*) sulphanilamide in 5 % (*v*/*v*) phosphoric acid and 0.1 % (*w*/*v*) naphthylethylenediamine-HCl] and incubated at room temperature for 15 min. The absorbance at 540 nm was then measured using a Victor-3 Multilabel counter (Perkin Elmer, Waltham, MA, USA). A standard curve was set in the culture medium by using serial dilutions of sodium nitrite (50 − 0.39 µM; linear regression: y = 0.0223x + 0.102; r = 0.99). The inter-assay variability was less than 5%.

### 2.5. RNA Extraction and Reverse Transcription (RT) 

Total RNA was isolated from about 1 × 10^6^ IPEC-J2/well and from about 1.5 × 10^7^ PBMC/well using TRI-reagent according to the manufacturer’s instructions and reverse-transcribed to generate complementary DNA (cDNA) using oligo-dT primers. Purity and concentration were assessed by using a BioSpectrometer^®^ (Eppendorf AG, Hamburg, Germany) at 260/280 and 260 nm, respectively. RNA samples were DNAse-treated and 1 µg/20 µL was reverse transcribed using a High-capacity cDNA Reverse Transcription kit. RT was performed using a StepOne thermocycler (Applied Biosystems, StepOne software v.2.3, Foster City, CA, USA) and, according to the manufacturer’s instructions, under the following thermal conditions: 10 min at 25 °C, 120 min. at 37 °C followed by 5 min at 85 °C. The cDNA samples were stored at −20 °C.

### 2.6. Real-Time PCR

The cDNA samples were used as a template for real-time quantitative PCR (qPCR) performed by using a StepOne thermocycler. The cDNA (20 ng/20 µL) was amplified in duplicate with the Fast Power-Up SYBR Green Master Mix along with specific sets of primers at 300 nM. The specifics of each primer set for identification of cytokine gene expression are reported in Table 1. Samples were kept at 95 °C for 20 s (hold step) to allow DNA-polymerase activation and were then subjected to 40 cycles consisting of a denaturation step at 95 °C for 3 s followed by an annealing/extension step at 60 °C for 30 s. Fluorescence due to SYBR Green incorporation was acquired at the end of the extension step. The reference *glyceraldehyde-3-phosphate dehydrogenase* (*GAPDH*) gene was selected among the other tested reference genes (i.e., *β-2-microglobulin* (*β-2MG*) [16], *hypoxanthine phosphoribosyltransferase-1* (*HPRT-1*) [17], and *18S rRNA* [18]) as the endogenous control according to minimal intra-/inter-assay variation and according to Facci et al. [19] and Ferrari et al. [20]. Data were analysed according to the 2^−ΔΔCt^ method [21] in which the expression levels of each cytokine, normalized to the *GAPDH* cDNA amount and expressed as relative quantities (RQ), were calculated with regards to the expression level in IPEC-J2 or PBMC monoculture conditions in DMEM/Ham’s F-12 medium at 24 h. A melting curve analysis for specific amplification control was performed (60–95 °C) at the end of the amplification cycles. No-RT controls and no-template controls (NTC) were included and the latter were assumed to be negative and reliable if the quantification cycle (Cq) was ≥35.

### 2.7. Statistical Analysis

Each experiment was repeated six times and each culture condition was performed with eight replicate wells. Data were analysed by ANOVA (IBM^®^ SPSS^®^ Statistics v.26, Armonk, NY, USA) using a model with group, sampling time and interaction between group and sampling time as fixed factors. The least significant difference (LSD) post-hoc test was used to compare means when significant differences (*p* < 0.05) were found. Experimental data were presented as least squares means ± standard error (SE). Differences among groups were considered significant if *p* < 0.05.

## 3. Results

### 3.1. Cell Viability

**Figure 1:**
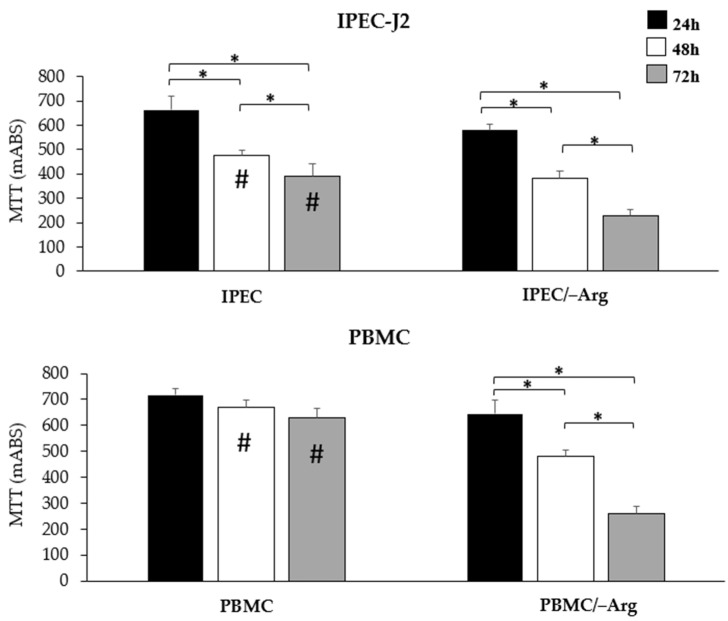
Cell viability of IPEC-J2 and PBMC determined using an MTT assay at 24, 48 and 72 h of incubation. Significant differences (*p* < 0.05) at 24, 48 or 72 h in the same group are indicated with hashtags. Significant differences (*p* < 0.05) among groups at the same time-point are indicated with asterisks.

Arginine deprivation in the culture medium (IPEC/–Arg group) led to lower cell viability in IPEC-J2 cells compared to controls (IPEC group) (Figure 1). At 48 h of incubation, cell viability significantly decreased in both groups compared to at 24 h (*p* < 0.05). The absence of arginine exhibited enhanced cytotoxicity in a time-dependent manner and, at 72 h of incubation, IPEC-J2 showed a drastic reduction of viability (*p* < 0.05). PBMC in the arginine-free medium (PBMC/–Arg group) showed a comparable trend with IPEC-J2 (groups IPEC and IPEC/–Arg) while PBMC in the complete medium (PBMC group) did not show a significant reduction in viability over time.

### 3.2. Nitric Oxide Accumulation

**Figure 2:**
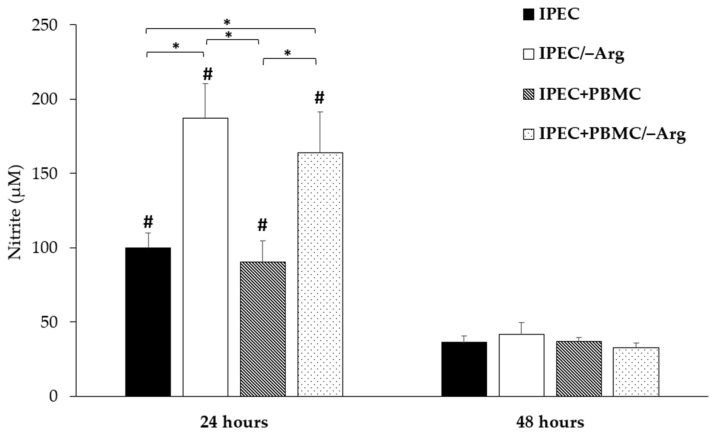
Effects of the culture condition (absence of arginine in mono and co-culture) on nitrite release at 24 and 48 h of incubation. Each values represents the mean ± SE of 8 wells of 6 independent experiments. Significant differences (*p* < 0.05) among groups are labelled with asterisks. Significant differences (*p* < 0.05) between 24 and 48 h in the same group are indicated with hashtags.

Figure 2 shows the significant effect of arginine deprivation on NO accumulation in the culture medium at 24 h (*p* < 0.05): the highest response was observed in the IPEC/–Arg group in which the NO value showed a two-fold increase as compared to the control group (IPEC). In the co-culture system, arginine deprivation also induced a strong NO release (IPEC+PBMC/–Arg group) (*p* < 0.05). At 48 h of incubation, the amount of NO was significantly reduced in all groups (*p* < 0.05).

### 3.3. Gene Expression

#### 3.3.1. *Cationic Amino Acid Transporter-1* (*CAT-1*)

**Figure 3:**
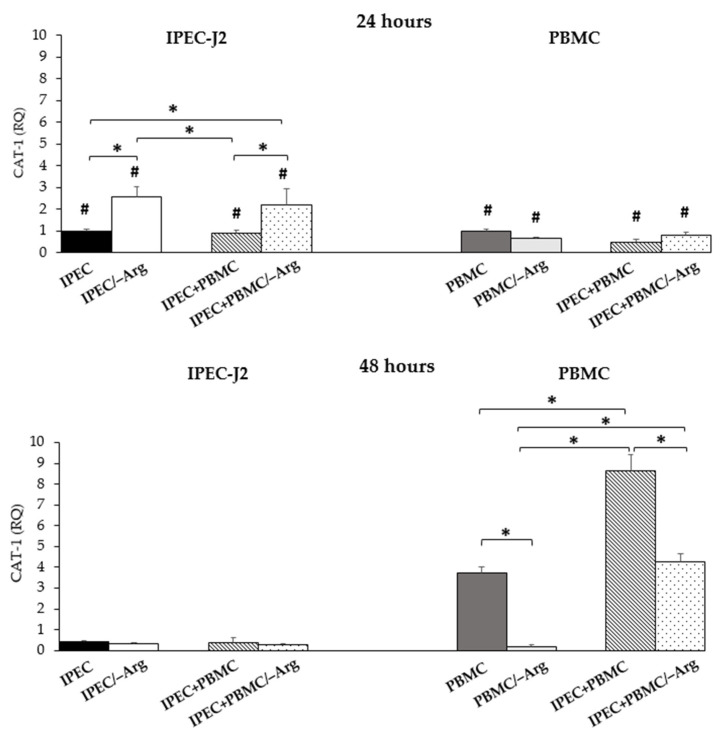
*Cationic Amino Acid Transporter-1* (*CAT-1*) gene expression in IPEC-J2 cells and in PBMC at 24 and 48 h of incubation. Each value represents the mean ± SE of 8 replicates of 6 independent experiments. Significant differences (*p* < 0.05) among groups (upon row comparisons within paired groups) are indicated with asterisks. Significant differences (*p* < 0.05) between 24 and 48 h in the same group (upon column comparisons vs. respective control group) are indicated with hashtags. Data were analysed according to the 2^−ΔΔCt^ method, in which the expression levels of each cytokine, normalized to the *GAPDH* cDNA amount, were expressed as relative quantities (RQ).

In IPEC-J2, *CAT-1* expression in the IPEC+PBMC group was not different from the control group (IPEC) at 24 h of culture. Groups IPEC/–Arg and IPEC+PBMC/–Arg (arginine-deprived conditions) showed a significant increase (*p* < 0.05) compared to the control (IPEC group). A significant decrease of expression was detected in the presence of arginine in groups IPEC and IPEC+PBMC, as compared to groups IPEC/–Arg and IPEC+PBMC/–Arg. At 48 h of incubation, no significant differences were observed among groups. *CAT-1* expression in PBMC at 24 h was not influenced by the culture condition (presence or absence of arginine), while arginine deprivation in the PBMC monoculture (PBMC/–Arg group) at 48 h induced a strong reduction of expression as compared to the control group (PBMC) (*p* < 0.05). In the co-culture condition at 24 h, the lack of arginine (IPEC+PBMC/–Arg group) did not influence *CAT-1* expression compared to the monoculture control (PBMC group). At 48 h, in the IPEC+PBMC group (co-culture in complete medium), *CAT-1* expression strongly increased as compared to groups PBMC (control) and IPEC+PBMC/–Arg. At 48 h, *CAT-1* expression in IPEC-J2 decreased in all groups, whereas it highly increased in PBMC in all conditions except for the PBMC/–Arg group compared to the values at 24 h (*p* < 0.05). Data are shown in Figure 3.

#### 3.3.2. *Tumour Necrosis Factor-α* (*TNF-α*)

**Figure 4:**
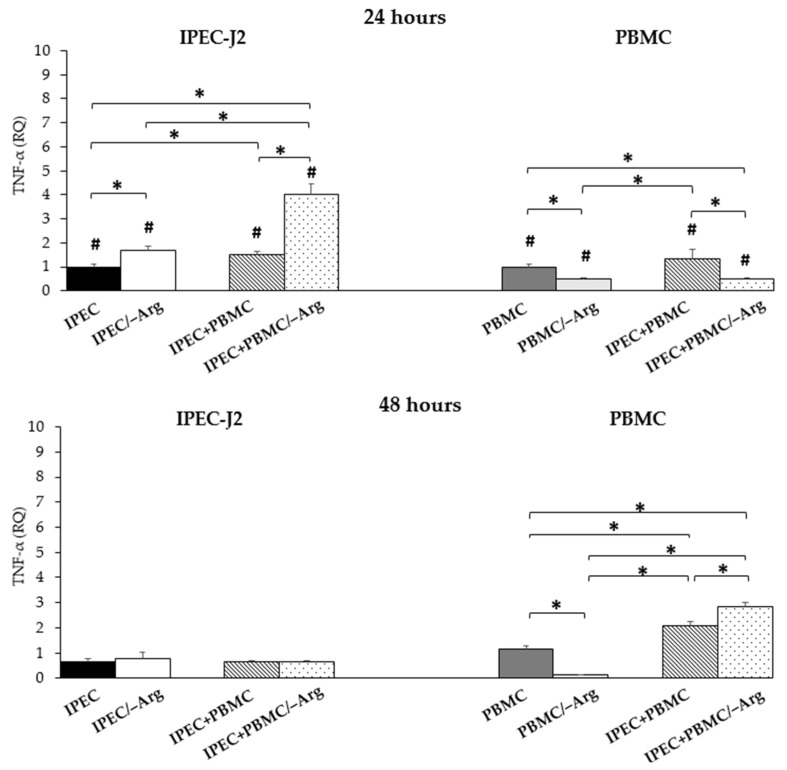
*Tumour Necrosis Factor-α* (*TNF-α*) gene expression in IPEC-J2 cells and in PBMC at 24 and 48 h of incubation. Each value represents the mean ± SE of 8 replicates of 6 independent experiments. Significant differences (*p* < 0.05) among groups (upon row comparisons within paired groups) are indicated with asterisks. Significant differences (*p* < 0.05) between 24 and 48 h in the same group (upon column comparisons vs. respective control group) are indicated with hashtags. Data were analysed according to the 2^−ΔΔCt^ method, in which the expression levels of each cytokine, normalized to the *GAPDH* cDNA amount, were expressed as relative quantities (RQ).

At 24 h of incubation, *TNF-α* expression in IPEC-J2 was significantly enhanced by arginine deprivation (IPEC/–Arg group) compared to the control (IPEC group) (*p* < 0.05), as shown in Figure 4. The co-culture condition positively modulated *TNF-α* expression (groups IPEC+PBMC and IPEC+PBMC/–Arg) (*p* < 0.05) as compared to the control group (IPEC). In particular, the highest expression was observed in the absence of arginine (IPEC+PBMC/–Arg group) as compared to groups IPEC and IPEC+PBMC. In all groups, the expression decreased at 48 h of incubation. In PBMC, *TNF-α* expression was down-regulated by the lack of arginine at 24 h in groups PBMC/–Arg and IPEC+PBMC/–Arg compared to the control (PBMC group) (*p* < 0.05). The expression in the IPEC+PBMC group was higher as compared to groups IPEC/–Arg and IPEC+PBMC/–Arg, while no differences were detected in the IPEC+PBMC group compared to the PBMC group (control). The expression in PBMC at 48 h of co-culture increased up to 1.7 and 2.4 fold in groups IPEC+PBMC and IPEC+PBMC/–Arg, respectively, compared to controls (PBMC group) (*p* < 0.05). In the IPEC+PBMC/–Arg group, *TNF-α* was significantly (*p* < 0.05) higher as compared to the IPEC+PBMC group. The absence of arginine inhibited *TNF-α* expression in the PBMC/–Arg group also at 48 h (*p* < 0.05). At this time-point, the PBMC/–Arg group showed the lowest values in comparison with the other groups. At 48 h, *TNF-α* expression in IPEC-J2 decreased in all groups whereas in PBMC, co-culture values increased compared to at 24 h (*p* < 0.05). Concomitantly, the expression in the PBMC/–Arg group decreased (*p* < 0.05). Data are shown in Figure 4.

#### 3.3.3. *Interleukin-1α* (*IL-1α*)

**Figure 5:**
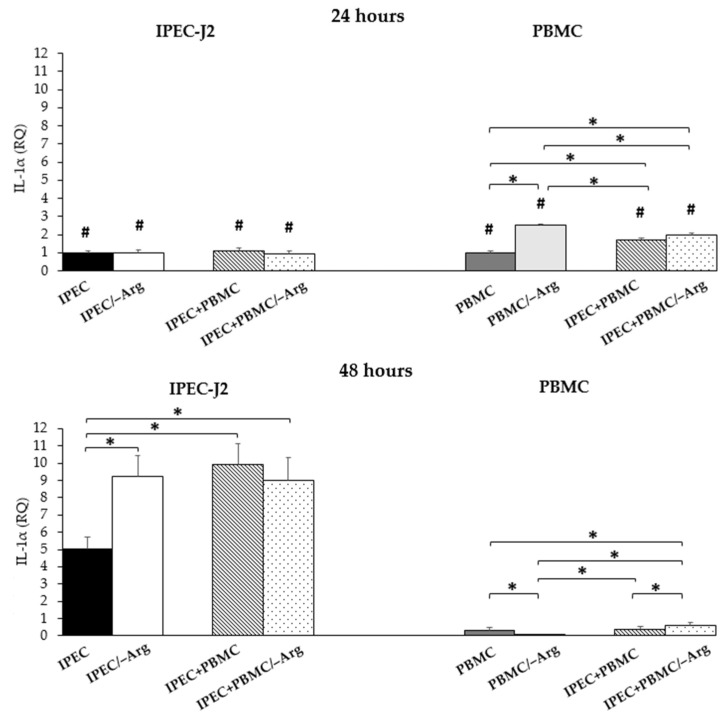
*Interleukin-1α* (*IL-1α*) gene expression in IPEC-J2 cells and in PBMC at 24 and 48 h of incubation. Each value represents the mean ± SE of 8 replicates of 6 independent experiments. Significant differences (*p* < 0.05) among groups (upon row comparisons within paired groups) are indicated with asterisks. Significant differences (*p* < 0.05) between 24 and 48 h in the same group (upon column comparisons vs. respective control group) are indicated with hashtags. Data were analysed according to the 2^−ΔΔCt^ method, in which the expression levels of each cytokine, normalized to the *GAPDH* cDNA amount, were expressed as relative quantities (RQ).

As shown in Figure 5, no differences of *IL-1α* expression in IPEC-J2 were observed at 24 h in all groups. In IPEC-J2 cells at 48 h of culture, *IL-1α* showed a significant increase in groups IPEC/–Arg, IPEC+PBMC and IPEC+PBMC/–Arg compared to the control (IPEC group) (*p* < 0.05). No significant differences among groups IPEC/–Arg, IPEC+PBMC and IPEC+PBMC/–Arg were observed. At 24 h, the expression in PBMC was stimulated in all experimental groups as compared to the control (PBMC group) (*p* < 0.05). In particular, the absence of arginine strongly influenced *IL-1α* expression (PBMC/–Arg group), as compared to groups IPEC+PBMC and IPEC+PBMC/–Arg. This condition at 48 h inhibited the expression in monoculture PBMC (PBMC/–Arg) as compared to the PBMC group (control), as well as groups IPEC+PBMC and IPEC+PBMC/–Arg). At this time-point, in co-culture conditions, the IPEC+PBMC/–Arg group showed an increase of IL-1α expression compared to the PBMC/–Arg group (*p* < 0.05) and to the co-culture group in the complete medium (IPEC+PBMC). This latter group showed no significant difference to the control (PBMC). *IL-1α* expression at 48 h in IPEC-J2 was enhanced in all groups compared to at 24 h (*p* < 0.05), whereas in PBMC a drastic drop occurred in all conditions (*p* < 0.05).

#### 3.3.4. *Interleukin-6* (*IL-6*)

**Figure 6:**
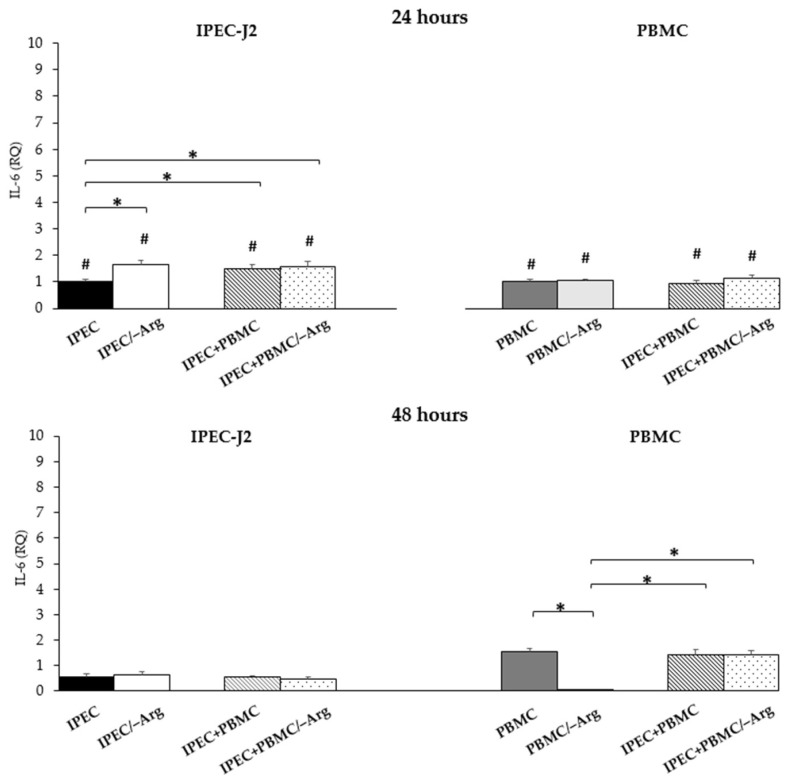
*Interleukin-6* (*IL-6*) gene expression in IPEC-J2 cells and in PBMC at 24 and 48 h of incubation. Each value represents the mean ± SE of 8 replicates of 6 independent experiments. Significant differences (*p* < 0.05) among groups (upon row comparisons within paired groups) are indicated with asterisks. Significant differences (*p* < 0.05) between 24 and 48 h in the same group (upon column comparisons vs. respective control group) are indicated with hashtags. Data were analysed according to the 2^−ΔΔCt^ method in which the expression levels of each cytokine, normalized to the *GAPDH* cDNA amount, were expressed as relative quantities (RQ).

Figure 6 shows the significant increase of *IL-6* expression in groups IPEC/–Arg, IPEC+PBMC and IPEC+PBMC/–Arg at 24 h of culture compared to the control (IPEC group) (*p* < 0.05). No significant differences were observed among the three groups. At 48 h, *IL-6* decreased in IPEC-J2 under all conditions compared to the values at 24 h (*p* < 0.05). At this time in IPEC-J2, none of the culture conditions (presence/absence of arginine; mono-/co-culture) affected *IL-6* expression. In PBMC, the presence or absence of arginine (groups PBMC/–Arg and IPEC+PBMC/–Arg) did not affect *IL-6* expression at 24 h of culture as compared to the control (PBMC). At 48 h incubation, *IL-6* expression increased in all groups, except for the PBMC/–Arg group, in which expression drastically decreased (*p* < 0.05). In particular, the rise of *IL-6* expression in groups IPEC+PBMC and IPEC+PBMC/–Arg was not statistically different as compared to the control (PBMC). At 48 h, the expression increased in all groups except for the PBMC/–Arg group compared to at 24 h (*p* < 0.05).

#### 3.3.5. *Interleukin-8* (*IL-8*)

**Figure 7:**
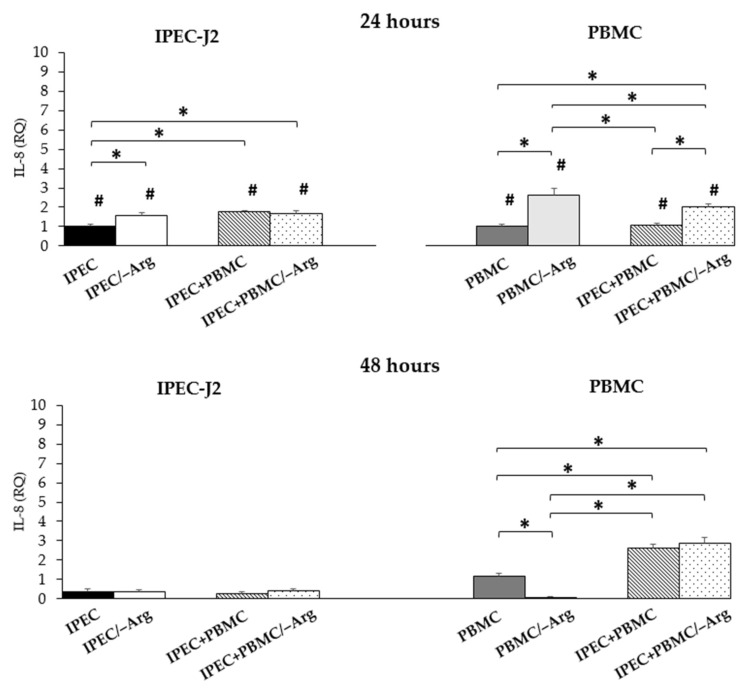
*Interleukin-8* (*IL-8*) gene expression in IPEC-J2 cells and in PBMC at 24 and 48 h of incubation. Each value represents the mean ± SE of 8 replicates of 6 independent experiments. Significant differences (*p* < 0.05) among groups (upon row comparisons within paired groups) are indicated with asterisks. Significant differences (*p* < 0.05) between 24 and 48 h in the same group (upon column comparisons vs. respective control group) are indicated with hashtags. Data were analysed according to the 2^−ΔΔCt^ method, in which the expression levels of each cytokine, normalized to the *GAPDH* cDNA amount, were expressed as relative quantities (RQ).

A significant increase of *IL-8* expression in IPEC-J2 cells was observed at 24 h of incubation in groups IPEC/–Arg and IPEC+PBMC compared to the control (IPEC group), while the IPEC+PBMC/–Arg group showed a significant increase compared to the IPEC group (*p* < 0.05) and no difference compared to the IPEC/–Arg group. At 48 h of incubation, a significant decrease was shown in all groups (*p* < 0.05) compared to the control (IPEC) at 24 h, without significant differences among groups. With regards to PBMC, the arginine-free condition significantly induced *IL-8* expression (groups PBMC/–Arg and IPEC+PBMC/–Arg) at 24 h of culture (*p* < 0.05), as compared to the control (PBMC). The highest expression was observed in the PBMC/–Arg group as compared to the IPEC+PBMC/–Arg group. No significant differences were found in the IPEC+PBMC group as compared to the control (PBMC). At 48 h, we observed an increase in groups IPEC+PBMC and IPEC+PBMC/–Arg compared to the control (PBMC group) (*p* < 0.05). At 48 h, *IL-8* expression was suppressed in PBMC seeded without arginine (PBMC/–Arg group) (*p* < 0.05). At the same time-point, *IL-8* increased in co-culture conditions whereas it drastically dropped in the PBMC/–Arg group (*p* < 0.05). Data are shown in Figure 7.

#### 3.3.6. *Transforming Growth Factor-β* (*TGF-β*)

**Figure 8:**
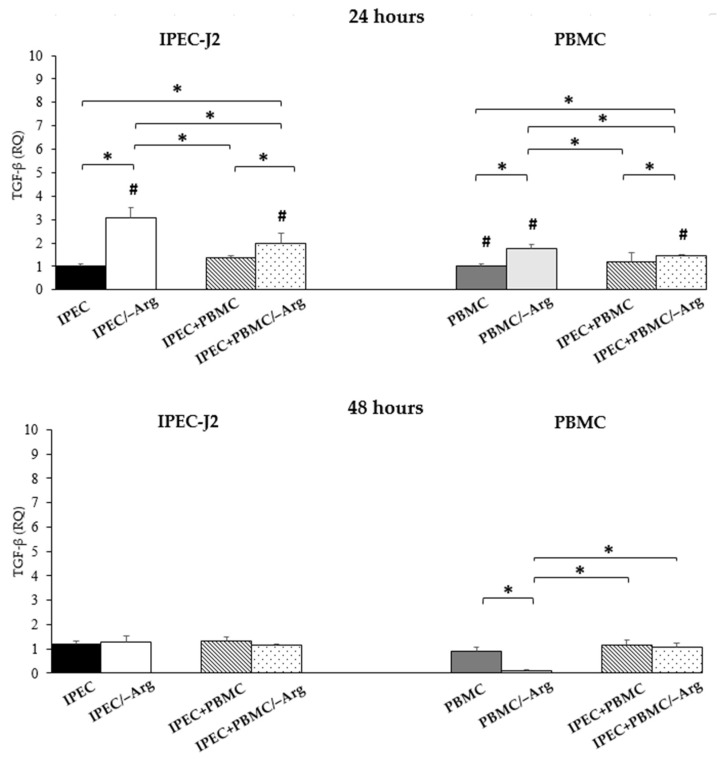
*Transforming Growth Factor-β* (*TGF-β*) gene expression in IPEC-J2 cells and in PBMC at 24 and 48 h of incubation. Each value represents the mean ± SE of 8 replicates of 6 independent experiments. Significant differences (*p* < 0.05) among groups (upon row comparisons within paired groups) are indicated with asterisks. Significant differences (*p* < 0.05) between 24 and 48 h in the same group (upon column comparisons vs. respective control group) are indicated with hashtags. Data were analysed according to the 2^−ΔΔCt^ method, in which the expression levels of each cytokine, normalized to the *GAPDH* cDNA amount, were expressed as relative quantities (RQ).

The expression of *TGF-β* in IPEC-J2 significantly increased in arginine deprivation conditions (IPEC/–Arg and IPEC+PBMC/–Arg) (*p* < 0.05), as compared to the control (IPEC). In particular, the IPEC/–Arg group showed a significantly higher expression as compared to the IPEC+PBMC/–Arg group. The IPEC+PBMC group was not different from the control group (IPEC). At 48 h of incubation, no significant differences among groups were observed. Likewise, PBMC showed the highest expression at 24 h of culture in groups PBMC/–Arg and IPEC+PBMC/–Arg (*p* < 0.05), as compared to control (PBMC). In the PBMC/–Arg group, the expression of *TGF-β* resulted significantly increased as compared to the IPEC+PBMC/–Arg group. At 48 h, the expression of *TGF-β* decreased in the PBMC/–Arg group compared to control (PBMC group) (*p* < 0.05). No significant differences were found between the other groups. In IPEC-J2, upon arginine deprivation, *TGF-β* significantly decreased compared to at 24 h (*p* < 0.05). In PBMC, 48-h incubation induced a strong *TGF-β* reduction in the absence of arginine (PBMC/–Arg and IPEC+PBMC/–Arg) (*p* < 0.05). Data are shown in Figure 8.

## 4. Discussion

The present work investigated a cell model to study the interplay between intestinal epithelial cells and immune cells in swine; considering the complexity of the gut system, this makes the in vitro approach intriguing and useful. IPEC-J2 cells have long been recognized as one of the specific models for the study of Intestinal Epithelial Cells’ (IECs) function in swine [15]. The IECs, in addition to their role as physical barrier, also participate directly and indirectly in the immune response by way of the cytokine and chemokine pathways [27]. In swine, a proper local defence reduces the risk of infection and disease, especially in the early stages of life when stress conditions (e.g., weaning, social interactions, changing feed) occur.

Arginine is a semi-essential amino acid, which becomes essential in young animals where the need exceeds synthesis [28]. In different species, it plays a protective role in IEC [29,30] and, in swine, arginine is known to be essential for newborns and piglets [31]. Arginine supplementation reduces intestinal damage and improves intestinal immunity in weaned piglets [32], but the underlying mechanism of its effect is largely unknown [33]. In our model, the absence of arginine strongly influenced viability/proliferation. The MTT assay results showed that the absence of arginine affects the proliferation of IPEC-J2 cells after both 24 and 48 h. When observed at 72 h, the cells cultured in the arginine-free medium died. As it can be clearly deduced, arginine also plays a fundamental role in PBMC: in fact, after 48 h of arginine deprivation, PBMC lose the ability to express most of the cytokines evaluated. Arginine is a precursor of amino acids and proteins [34], and its anabolic action is mediated by the mammalian target of rapamycin (mTOR), involved in one of the major anabolic cellular signalling patterns [35]. Arginine uptake is regulated by members of the CAT family [36]; in particular, *CAT-1* is ubiquitous in most cells and is the main arginine transporter. In our study, *CAT-1* expression was affected by arginine supplementation. In fact, in the presence of arginine (groups IPEC and IPEC+PBMC), the expression of *CAT-1* in IPEC-J2 seemed to reflect the availability of arginine in that it was higher at 24 h than at 48 h. The decrease may be related to the reduction of available arginine in the medium. Groups IPEC/–Arg and IPEC+PBMC/–Arg (arginine-deprived conditions) showed a similar trend, but with a much higher expression at 24 h compared to the control (IPEC group). This was an unexpected result, but we hypothesize that the reduction of arginine in the cells could activate an ex-novo synthesis from precursors such as glutamate, which is a constitutive element of the media used. In fact, at the small intestine level, glutamine and glutamate are major precursors for the intestinal synthesis of arginine [37]. Another means of arginine synthesis involves the conversion of ornithine, which is not present in the formulation of the culture medium, but it derives from the conversion of glutamate [38]. The ex-novo synthesis could be perceived as a new availability of arginine and thus induce a positive feedback on *CAT-1* expression to overcome arginine intracellular deficiency. This hypothesis is also supported by the increase of nitrite accumulation in the arginine-free culture media (groups IPEC/–Arg and IPEC+PBMC/–Arg), since nitric oxide is generated by arginine [39]. After 48 h, *CAT-1* expression significantly decreased, concurrently to the decrease of nitrite. Arginine availability and, consequently, *CAT-1* expression in IPEC-J2 were also influenced by the presence of PBMC. Arginine positively modulates the metabolic activity and survival capacity of PBMC [40]. In the co-culture model, *CAT-1* expression in PBMC was not modulated by arginine deprivation and/or by the co-culture condition at 24 h. We can suppose that, at this time-point, PBMC still have availability of the arginine present in the medium used for stimulation with PHA. We know that the reduction in the intracellular levels of arginine occurs between 24 and 48 h after activation [40]. The increase of *CAT-1* after 48 h may confirm this hypothesis: in co-culture conditions (IPEC+PBMC and IPEC+PBMC/–Arg), the increased *CAT-1* expression should lead to the recovery of the arginine still potentially available in the medium and necessary for cell survival. The role of arginine is evident also by the evaluation of cytokine gene expression in IPEC-J2. Intestinal epithelial cells function as a line of defence by forming a physical barrier, but also by secreting pro-inflammatory cytokines and chemokines [3,27,41]. The secretion of pro-inflammatory mediators by IEC is two-faced and the mechanisms regulating intestinal inflammatory responses must be specific to avoid uncontrolled responses leading to chronic inflammation [42]. In agreement with other studies characterising this cell line [15,25], our previous unpublished data confirmed the lack of expression of *IL-1β*, *IL-10* and *IL-4* by IPEC-J2. Conversely, the expression of pro-inflammatory cytokines (*TNF-α*, *IL-1α*, *IL-6* and *IL-8*) was observed and influenced both by the presence of PBMC and by the stressful nutritional condition. TNF-α plays a pivotal role in starting the inflammatory cascade [43,44] and it controls multiple cellular processes such as the production of inflammatory mediators and proliferation. Local TNF-α is secreted by the intestinal epithelium itself during intestinal inflammation [27,45] and IECs also express TNF-α receptor isoforms that transduce intracellular signalling [46]. In our study, *TNF-α* expression in IPEC-J2 at 24 h was significantly higher in groups IPEC/–Arg, IPEC+PBMC and IPEC+PBMC/–Arg compared to the control. In particular, the presence of PHA-activated PBMC seems to intensify cell responsiveness to arginine deprivation. Our data highlight two aspects: first, arginine deprivation is a stressful stimulus that is able to induce a direct pro-inflammatory response, confirming the recognized essential role of this amino acid [47,48]; secondly, our system, allowing the crosstalk between epithelial cells and PBMC, points out the close collaboration and reciprocal influence between the two cell systems. These aspects are already clearly evident from the analysis of the different groups after 24 h. The behaviour of IPEC-J2 in the absence of arginine (IPEC/–Arg group) highlights the direct response capacity to the stimulus. The increase in *TNF-α* expression in the IPEC+PBMC group (co-culture in the complete medium) instead indicates the reciprocal effect between PBMC and IPEC-J2: the expression in IPEC-J2 is in fact significantly greater than in the control group. PBMC behave in the same way: *TNF-α* expression was greater than that in monoculture PBMC. In this condition, we noted a mutual influence between the two cell types which results in an increase in *TNF-α* expression in PBMC at 48 h. The IPEC+PBMC/–Arg group emphasizes this crosstalk even more: in fact, we reported that in the IPEC+PBMC/–Arg group, the increase of *TNF-α* in PBMC is sequential (48 h) to that of IPEC-J2 (24 h). The IECs’ response could have induced an up-regulation in PBMC expression to sustain the cellular response to the negative metabolic stimulus. In fact, as TNF-α is known to promote the additional TNF-α cascade [49], we may also suppose an autocrine action through its receptors. The role as response trigger is also confirmed in all groups at 48 h when the expression of the cytokine drops drastically, returning to the control values. This result is also influenced by the reduction in cell viability: at 48 h of incubation, we also observed a reduction of expression in the control group (IPEC), supported by the reduction of cell viability. Indeed, TNF-α controls the production of other inflammatory mediators, such as IL-1α, and IL-8. IL-1α functions as an “alarmin” following cell death signals [50]. Apoptosis signals move IL-1α from the cytosol to the nucleus where, by binding to chromatin, it is not available to start the inflammatory process. Only following a necrosis signal does IL-1α leave the nucleus to initiate the inflammatory response [51]. *IL-1α* showed an increase in groups IPEC/–Arg, IPEC+PBMC and IPEC+PBMC/–Arg at 48 h, confirming an influence on its modulation by *TNF-α*, associated with the signals of reduction in cell viability. This hypothesis is supported by the increase in *IL-1α* levels in the IPEC group as well. The cytokine was also expressed by PBMC and we detected an increase at 24 h, probably related to the simultaneous increase of *TNF-α* in IPEC-J2. IL-6 also plays the role of warning signal and its expression quickly increases in response to local stress factors. *IL-6* gene transcription is modulated by a great number of transcription factors, including TNF-α [52]. In our model, IPEC-J2 showed an increase of *IL-6* expression in groups IPEC/–Arg, IPEC+PBMC and IPEC+PBMC/–Arg at 24 h, but, similarly to *TNF-α*, *IL-6* decreased at 48 h. IL-6 is a pleiotropic cytokine with high clinical relevance and an important mediator of cellular communication, orchestrating both pro- and anti-inflammatory processes [53]. IL-6-induced signalling is initiated by binding to IL-6 receptor α, which exists in a soluble and in a transmembrane form. Binding of IL-6 to its membrane-bound receptor induces anti-inflammatory classic signalling, whereas binding of IL-6 to the soluble receptor induces pro-inflammatory trans-signalling [54]. The trend of *IL-6* could reflect the activation of this latter pathway: the trans-signalling guarantees the pro-inflammatory response in synergy with the other pro-inflammatory cytokines, as shown by the rise in *IL-6* expression after 24 h. TNF-α also regulates IL-8 production [55], a chemokine capable of recruiting neutrophils and immune cells to the infection site [43]. In the differentiated human cell line CACO-2, TNF-α induces apical and basolateral IL-8 secretion and the mechanism is mediated by type 1 and type 2 TNF-α receptors [56]. In our study, *IL-8* and *TNF-α* showed an analogous expression pattern in groups IPEC/–Arg, IPEC+PBMC and IPEC+PBMC/–Arg, with a rise in gene expression after 24 h and a decrease at 48 h. In our model, *IL-8* expression in PBMC seems to confirm this role, showing the same expression patterns as *TNF-α*. Arginine seems to have a critical role in the modulation of pro-inflammatory cytokines, likely due to its ability to increase anti-inflammatory factors [33]. We know that TGF-β is abundant in the intestine and it is also produced by epithelial cells [57,58]. TGF-β1 is an important factor in intestinal epithelial cell restitution [59] and its expression and production have been shown to be stimulated by arginine supplementation [33]. On the basis of the role of TGF-β in gut immune cell homeostasis [60,61] the evaluation of *TGF-β* expression allowed us to better characterize the response of IPEC-J2 to the stressful condition. In fact, the *TGF-β* expression levels in IPEC-J2 appeared to be strictly influenced by the absence of arginine after 24 h. Our data apparently contrasted with the results reported by Wu et al. [33]. In fact, we incubated the cells for longer durations (24 or 48 h vs. 12 h) and the rise in *TGF-β* expression after 24 h in deprived groups is in line with the hypothesis of a new arginine availability from the ex-novo synthesis.

## 5. Conclusions

In the present investigation, we focused on the study of the in vitro cooperation between activated immune cells and intestinal epithelial cells during a stressful stimulus (deprivation of an essential amino acid).

Our preliminary data determined that:

- arginine deficiency strongly influences IECs and stimulates their functional response;
- the IPEC-J2/PBMC co-culture model is functional to elucidate the interaction between IEC and immune cells;
- in order to better characterize this model, it will be necessary to investigate how gene expression is associated/correlated with the secretion of these and other mediators involved in the cross-talk between intestinal cells and immune cells.

Further studies will be important to clarify the role of arginine in IPEC-J2 as a modulator of the local anti-inflammatory response to adapt and react to a stress-inducing stimulus. We think that the application of this experimental model could be important for studying and clarifying the role of nutritional conditions in IEC functionality and responsiveness in swine and also in humans based on the similarities between pigs and humans in terms of gut microbiota and in the mechanisms of the intestinal defence systems. In order to assess these aspects, isolated resident immune cells of the intestine, such as intraepithelial lymphocytes (IEL), lymphocytes of the Peyer’s patches (PP) and lamina propria (LP) immune cells—which constitute the intestinal mucosal immune system—can be employed in co-culture conditions with IPEC-J2 to properly simulate in vivo conditions both spatially and functionally. Together, this approach supports the feasibility of developing a system to investigate the impact of and the interaction between specific nutrients and the complex intestinal environment. With particular reference to swine, our cell co-culture model can be used to evaluate feed additives to improve animal health and production, identifying the interplay between IECs and immune cells in the protective response to stimuli.

## Figures and Tables

**Table 1:** Target genes and details of the primer sequences used for quantitative SYBR Green real-time PCR amplification. The *GAPDH* gene was used as the endogenous control gene.

Target Gene	GenBank Accession No	Primer Sequence	[C] (nM)	Efficiency (%)	Slope	r^2^	Amplicon Length (bp)
*CAT-1* [22]	NM_001012613	F: 5′-AGACGGGCTGCTGTTTAAGT-3′ R: 5′-ACCGTTAAAATACCGGCGTG-3′	300	100.6	−3.30	0.99	131
*IL-8* [23]	NM_213867	F: 5′-CCGTGTCAACATGACTTCCAA-3′ R: 5′-GCCTCACAGAGAGCTGCAGAA-3′	300	98.9	−3.29	0.99	75
*IL-6* [24]	NM_214399	F: 5′-GGCAAAAGGGAAAGAATCCAG-3′ R: 5′-CGTTCTGTGACTGCAGCTTATCC-3′	300	99.6	−3.33	0.99	87
*TGF-β1* [25]	NM_214015	F: 5′-AGGGCTACCATGCCAATTTCT-3′ R: 5′-CCGGGTTGTGCTGGTTGTACA-3′	300	106.3	−3.18	0.98	102
*TNF-α* [24]	NM_214022	F: 5′-ACTGCACTTCGAGGTTATCGG-3′ R: 5′-GGCGACGGGCTTATCTGA-3′	300	98.5	−3.36	0.99	118
*IL-1α* [26]	NM_214029	F: 5′-GCTCAAAACGAAGACGAACC-3′ R: 5′-TGATGGTTTTGGGTGTCTCA-3′	300	99.4	−3.34	0.97	61
*GAPDH* (Primer Express)	NM_001206359	F: 5′-GGTGAAGGTCGGAGTGAACG-3′ R: 5′-GCCAGAGTTAAAAGCAGCCCT-3′	300	102.0	−3.27	0.99	70

bp: base pairs; F: forward primer; R: reverse primer. *CAT-1*: cationic amino acid transporter-1; *IL*: interleukin; *TGF-β1*: transforming growth factor-beta 1; *TNF-α*: tumour necrosis factor-alpha; *GAPDH*: glyceraldehyde 3-phosphate dehydrogenase.

## Data Availability

The data presented in this study are available on request from the corresponding author.
